# Wide-Field Fluorescence Microscopy of Real-Time Bioconjugation Sensing

**DOI:** 10.3390/s18010290

**Published:** 2018-01-19

**Authors:** Marcin Szalkowski, Karolina Sulowska, Justyna Grzelak, Joanna Niedziółka-Jönsson, Ewa Roźniecka, Dorota Kowalska, Sebastian Maćkowski

**Affiliations:** 1 Faculty of Physics, Astronomy and Informatics, Nicolaus Copernicus University, Grudziadzka 5, 87-100 Torun, Poland; marszal@fizyka.umk.pl (M.S.); sulowska@fizyka.umk.pl (K.S.); justynag@fizyka.umk.pl (J.G.); dorota@fizyka.umk.pl (D.K.); 2 Baltic Institute of Technology, Al. Zwycięstwa 96/98, 81-451 Gdynia, Poland; 3 Institute of Physical Chemistry Polish Academy of Sciences, ul. Kasprzaka 44/52, 01-224 Warsaw, Poland; erozniecka@ichf.edu.pl

**Keywords:** fluorescence imaging, bioconjugation, plasmon enhancement, silver nanowire

## Abstract

We apply wide-field fluorescence microscopy to measure real-time attachment of photosynthetic proteins to plasmonically active silver nanowires. The observation of this effect is enabled, on the one hand, by sensitive detection of fluorescence and, on the other hand, by plasmonic enhancement of protein fluorescence. We examined two sample configurations with substrates being a bare glass coverslip and a coverslip functionalized with a monolayer of streptavidin. The different preparation of the substrate changes the observed behavior as far as attachment of the protein is concerned as well as its subsequent photobleaching. For the latter substrate the conjugation process is measurably slower. The described method can be universally applied in studying protein-nanostructure interactions for real-time fluorescence-based sensing.

## 1. Introduction

The development of nanotechnology, which has been very rapid and multidirectional for more than two decades [1], concerns primarily two major aspects. On the one hand, particular emphasis has been placed at developing ways to control the morphology of nanoobjects to enable tuned manipulation of electrical, optical or chemical properties thereof [2]. Another important facet of this research field is to assemble—also in a controlled way—hybrid functional nanostructures, where interactions between them can be tuned and exploited from the point of view of any required application. In particular, in the case of metallic nanostructures, free electrons upon interaction with incoming light can form an oscillation, called localized plasmon resonance (LPR) [3]. The energies of these resonances depend not only on the material, but are also very sensitive to the geometry (shapes and sizes) of the metallic nanoparticles [4]. The remarkable ability to concentrate electromagnetic radiation to dimensions comparable or less than the wavelength of light, renders the LPR a versatile tool for electromagnetic field engineering, including applications in surface enhanced Raman scattering (SERS) [5,6] or metal-enhanced fluorescence (MEF) [7]. A second effect is associated with an increase of emission intensity accompanied with shortening of the fluorescence lifetime of fluorophores interacting with plasmon excitations in metallic nanoparticles [8,9]. The strength of this interaction strongly depends on the distance between the fluorophore and the metallic nanoparticle, their spectral properties and relative orientation. When the emitter is placed at distance in the order of 10–30 nm, one can observe a dramatic increase of the fluorescence quantum yield, and thus the intensity can also be significantly increased due to MEF [9]. On the other hand, when placed too close to a metallic nanoparticle, the emission of the fluorophore can be completely quenched due to energy transfer to the metallic nanoparticle and subsequent dissipation of energy as heat [3,4,9]. 

The properties of metallic nanostructures, including plasmon-related effects, have been used for sensing proteins [10], viruses [11], or other species, by employing either changes in local dielectric environment [12], enhancement of Raman scattering efficiency [13] or improvement of emission intensity [14,15,16]. The MEF effect has been studied in a variety of geometries to observe emission of dyes, polymers, proteins, also in structures where the emitters were specifically bound to metallic nanoparticles [17,18,19]. At the same time, reports focusing on real-time monitoring of conjugation and thus plasmon-induced effects have been rare, despite the recent progress in large-area sensitive detectors. 

In this work we demonstrate the real-time fluorescence imaging of a hybrid nanostructure composed of silver nanowires and natural light-harvesting proteins. The silver nanowires were appropriately functionalized to facilitate specific attachment of Peridinin–Chlorophyll–Protein (PCP). The PCP complex has recently been used to probe plasmonic interactions in various nanostructures [20] due to its structural simplicity and solubility in water. Indeed, several geometrical configurations of hybrid nanostructures composed of metallic nanoparticles and photosynthetic complexes have been studied recently to demonstrate the MEF effect. These include a layer-by-layer structure where photosynthetic complexes are deposited on planar metallic surfaces [21,22], or a mixture of PCP complexes and suspension of metallic nanoparticles [23]. However, such architectures offer at most a limited ability of controlling key parameters that determine the strength of the interaction between proteins and metallic nanoparticles, in particular the distance and mutual orientation. Thus, in order to achieve a higher degree of control over the morphology, here we focus on bioconjugation, which, when successful, should yield a monolayer of proteins oriented homogeneously on the surface of the metallic nanowires. The key reason for using metallic nanowires is that on the one hand they promote plasmon excitations, which can be used for enhancing the optical response of emitters, and on the other hand, can be directly visualized using standard optical/fluorescence microscopy. In this way, by correlating the images we can precisely assign emerging emission with the positions of the nanowires. This would be much more difficult in the case of small metallic nanoparticles or metallic planar surfaces, except for the fact when such nanoparticles are ordered on a substrate, but it is rather advanced technologically to obtain such structures. 

The results obtained for such hybrid nanostructures indicate that wide-field florescence microscopy is a method of choice to monitor dynamics of conjugation that takes place between photosynthetic proteins and silver nanowires. We find that the attachment occurs within a few seconds, and exhibits dependence on the substrate preparation. This method could be universally applied for real-time biochemical sensing with metallic, plasmonically-active nanostructures or platforms, presumably down to single protein level.

## 2. Materials and Methods

Silver nanowires (AgNWs) were prepared as described previously [24] and stored in aqueous solution. As measured with scanning electron microscopy [23], their diameters are from 50 to 200 nm and lengths from a few to up to 60 microns. Prior to the experiments, several steps towards proper functionalization of AgNWs were introduced. First, water was removed by centrifugation and AgNWs were diluted in 0.25 mM solution of cysteamine in pure ethanol (HPLC grade). The suspension was then incubated for 2 h in order to activate coupling between AgNWs and cysteamine. Afterwards, both ethanol and unbound cysteamine molecules were removed with centrifugation, and AgNWs covered with cysteamine were diluted in 0.15 mM solution of NHS-biotin in dimethyl sulfoxide (HPLC grade). The resulting suspension was kept overnight to facilitate the binding of cysteamine with NHS-biotin. As the last step of the functionalization, dimethyl sulfoxide and unbound NHS-biotin were removed by centrifugation, and the final product of functionalized AgNWs was dispersed in pure water.

Peridinin–Chlorophyll–Protein (PCP) is a water-soluble light-harvesting complex, which consists of eight peridinin molecules and two Chlorophyll a molecules embedded in a protein scaffold [25]. In this work we used PCP complexes from *Glenodinium* species of dinoflagellates, functionalized with streptavidin molecules (BD-Biosciences). 

Absorption spectrum of PCP and extinction spectrum of AgNW aqueous suspension were measured using Varian Cary 50 spectrophotometer, while fluorescence spectrum of PCP was taken with Jobin Yvon Fluorolog 3 spectrofluorimeter. Fluorescence maps and movies were collected using a wide-field fluorescence microscope working in a back-scattering geometry. It is based on a Nikon Ti-U inverted microscope body. Excitation (λ = 405 nm, FWHM = 11.49 nm, P = 40 µW) was provided by a LED illuminator (Prizmatix Ltd., Holon 5885849, Israel), and the excitation beam was reflected by a dichroic mirror (T650 LPXR, Chroma, Bellows Falls, Vermont, USA) to an oil-immersion objective Plan Apo (100x) with numerical aperture of 1.4. In order to reduce contribution of parasitic light, dielectric bandpass optical filters HQ675-20 (Chroma) and FELH650 (Thorlabs, Newton, USA) were used. Andor iXon3 EMCCD camera was used as a detector. Acquisition time of 0.1 s was applied, also for collecting movies composed of subsequent fluorescence maps. Collected signals were measured with Electron Multiplication gain (EMgain) of 100, and digitalized with 14-bit analog-to-digital converter. The experimental configuration allows for acquiring fluorescence images and transmission maps, with sizes around 100 µm × 100 µm. 

The experiment was carried out in two steps. First, 10 µL of AgNWs suspension was spin-coated on a glass coverslip and upon mounting the coverslip on a stage of the microscope, the positions of AgNWs were determined using transmission mode. Next, the microscope was switched to fluorescence imaging mode and 5 µL of PCP solution (concentration of 0.2 µg/mL) was dropped onto the sample surface with constant acquisition of fluorescence images with collection time of 0.1 s. The movies were collected for 5 min. In order to compare substrates with different functionalization, in addition to a bare glass substrate we also used a glass substrate covered with streptavidin molecules, which inhibit attachment of PCP complexes. It was done by silanization of the coverslip with (3-aminopropyltriethoxysilane, APTES) similarly to procedure described in reference [26]. Briefly, the coverslip was placed in a desiccator in the fumes of silane (container with 30 µL of APTES) and a catalyst (container with 10 µL of triethylamine), and left at room temperature for 2 h under an argon atmosphere. Next, the reagents were removed and the coverslip was left for another 48 h in the desiccator under an argon atmosphere for curing. The amine-terminated coverslip was modified with biotin (1 mg/mL in PBS pH = 7.4 and 0.8 mg/mL *N*-(3-dimethylaminopropyl)-*N*’-ethylcarbodiimide hydrochloride) via peptide bond formation. This step was followed by streptavidin interaction with biotin to obtain streptavidin-covered glass. 

## 3. Results and Discussion

**Figure 1:**
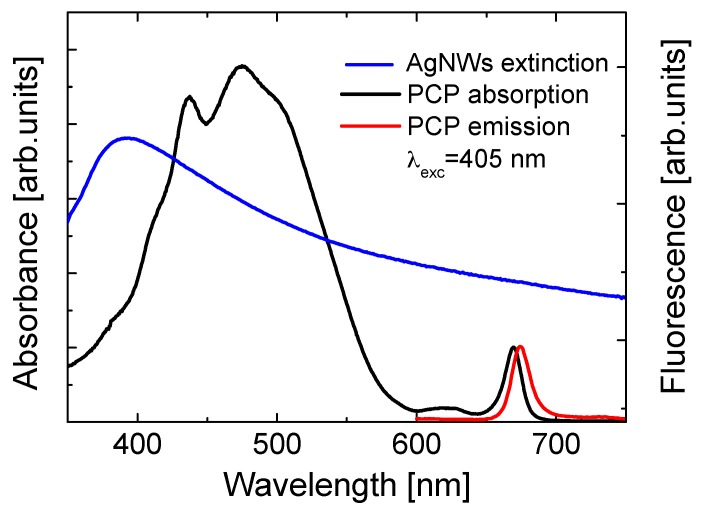
Absorption and emission spectra of Peridinin–Chlorophyll–Protein (PCP) (black and red lines, respectively) and extinction spectrum of AgNWs (blue line).

Absorption and fluorescence spectra of PCP, as well as the extinction spectrum of AgNWs, are presented in Figure 1. PCP absorption spectrum consists of a strong and broad band around 485 nm related to absorption of peridinins, which are the major absorbing molecules of PCP, and two bands, around 400 nm and 660 nm, related to the Soret and the Qy bands of Chlorophylls a, respectively. As the excitation from peridinins is efficiently (~90%, [25]) transferred to chlorophylls, the chlorophylls are responsible for radiative emission. It consists of a single peak with a maximum at 673 nm. The extinction spectrum of AgNWs is dominated by a broad peak with maximum at around 390 nm, resulting from plasmon excitations in AgNWs. Strong overlap between the optical spectra of the PCP complexes and AgNWs enables interactions between electronic states in the protein and plasmonic excitations in metallic nanostructures [23].

**Figure 2:**
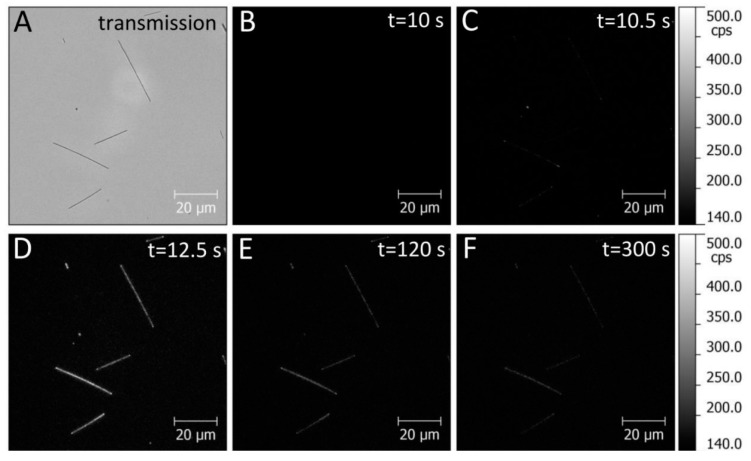
(**A**) Transmission image of silver nanowires (AgNWs) prior to deposition of the PCP solution, (**B**) fluorescence image of AgNWs prior to deposition of the PCP solution, (**C**–**F**) fluorescence maps of PCP emission acquired at indicated times. The intensity scale of all the images is identical, and the solution of PCP complexes was added at *t* = 10 s.

The real-time binding between streptavidin-functionalized PCP complexes and biotin-functionalized AgNWs was studied using wide-field fluorescence imaging. The movies collected for sample area of 100 µm × 100 µm (Supplementary Materials) were started for the substrates with AgNWs only (as proven using transmission mode of the microscope), and after 10 s 5 µL of PCP solution was dropped onto sample surface. The concentration of the protein used in the experiment (0.2 µg/mL) was to some degree optimized in order not to have too much protein attached to the surface off the nanowires, and secondly, to have enough protein for rather uniform coating of the nanowires. The movies were then analyzed and a series of images extracted from such a movie are displayed in Figure 2 together with the transmission image collected for the same sample area. The transmission image (Figure 2A) shows several nicely resolved nanowires, they are isolated from each other, enabling thus straightforward observation of the real-time attachment of proteins. Importantly, upon switching the microscope to the fluorescence detection mode (Figure 2B), we observe no emission of the nanowires themselves, indicating that functionalized AgNWs are optically dark in this spectral range. Adding of the solution of PCP complexes at *t* = 10 s resulted in rapid changes of fluorescence images. Namely, at locations of AgNWs the emission signal emerged, already after 0.5 s from depositing the protein solution (Figure 2C). With time the intensity of this emission rapidly increases, and it seems that after just a few seconds from the start of the conjugation, the fluorescence intensity reaches maximum values (Figure 2D). Importantly, we find essentially very little emission away from the AgNWs, which might be due to the PCP complexes that have fallen on the substrate. Subsequently, the emission exhibits gradual decrease in intensity, most probably due to photobleaching of chlorophyll molecules. The photobleaching however is found to be rather slow (Figure 2E,F) and even after 120 s and 300 s since the start of the experiment, the emission of the PCP complexes can be measured. An important feature of this set of images concerns perfect correlation between positions of AgNWs and the regions where the emission of the PCP complexes emerges upon adding the protein solution on the substrate. The effect of rapid brightening of PCP fluorescence at the positions identical with the location of the AgNWs can be explained by efficient binding between streptavidin-functionalized PCP complexes, and biotin molecules, which coat the AgNWs. Upon binding of more and more PCP complexes, the intensity of fluorescence emission increases up to the saturation point, when either no protein was available for conjugation, or no functional groups on AgNWs were available. From the fact that very little emission is seen in the areas off the nanowires, the former seems to be more probable. From this moment on, due to continuous illumination of the substrate, chlorophyll molecules start to undergo photobleaching, which is perhaps enhanced by simultaneous partial drying of the sample. As a result, the emission intensity decreases. 

The correlation between transmission and fluorescence images indicates that first of all, the PCP complexes attach specifically to the nanowires and that the emission is not quenched as a result of non-radiative energy transfer to metallic nanoparticles. This is important, as in the case of fluorescence quenching the approach used and described here would be inefficient. As in the case of PCP complexes the chlorophyll molecules responsible for fluorescence emission are shielded by the protein, the distance between them and silver nanowires is large enough to inhibit the non-radiative energy transfer. Thus, the approach of real-time monitoring of conjugation process seems to be highly suitable for functionalized and fluorescent proteins. 

**Figure 3:**
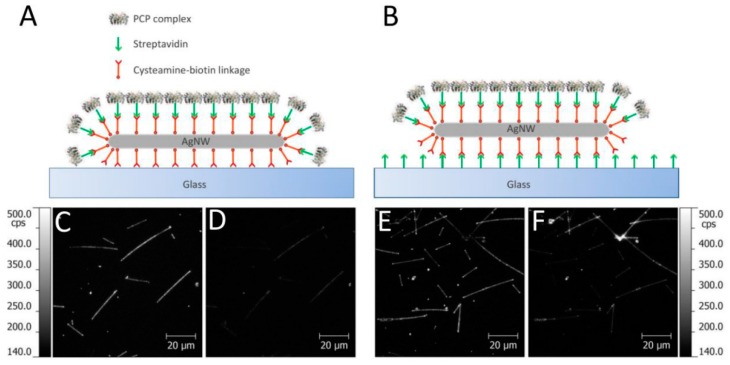
Schematic illustration of the structural differences between the two used substrates: bare glass (**A**) and streptavidin-covered glass (**B**). The fluorescence maps collected at 12.5 s (moment of maximum fluorescence intensity during the experiment) and at 300 s from the start of the measurement ((**C**,**D**), respectively) for bare glass substrate, and analogous fluorescence maps recorded at 30 s and 300 s from the start of the measurement for the streptavidin-covered glass ((**E**,**F**), respectively).

In order to study the influence of substrate preparation on the conjugation dynamics, we repeated the experiment for a glass substrate that was functionalized with streptavidin prior depositing silver nanowires. Such a substrate has two important functions: (1) attachment of AgNWs is much more efficient and robust, as the NWs are functionalized with biotin; and (2) it is essentially impossible for the PCP complex to fall on a substrate away from the AgNW. The structural difference between both substrates is presented schematically in Figure 3. In the case of a bare glass substrate, biotin molecules of the functionalized AgNWs are almost all available for conjugation with PCP complexes. In contrast, for the streptavidin-functionalized substrate part of biotin molecules covering AgNWs are used for attaching the nanowires to the substrate, thus the number of available functional groups for conjugation with the PCP complexes is less than in the previous case. As a result, we expect lower fluorescence intensities of PCP-AgNWs conjugates for this substrate, although at the same time, the contrast between the emission on and off the nanowires should be improved in comparison to a bare glass substrate. 

Dynamics of the conjugation process was measured for both types of substrates using the same approach as described previously. Figure 3C,D show fluorescence maps collected for a bare glass substrate at 12.5 s and at 300 s after starting the experiment, respectively. The fluorescence image acquired at 12.5 s corresponds to the maximum of fluorescence intensity measured during the whole sequence. After 300 s, a clear reduction of fluorescence intensity was observed. In contrast, for streptavidin-covered glass, maximum fluorescence intensity was measured after 30 s from the start of the experiment, which indicates that the conjugation process is much slower (Figure 3E). Furthermore, the PCP complexes conjugated to AgNWs placed on streptavidin-covered glass are much more photostable against illumination. It is evidenced by the fluorescence image taken after 300 s (Figure 3F), where the fluorescence intensity is much higher that for the corresponding image measured for a bare glass substrate. 

**Figure 4:**
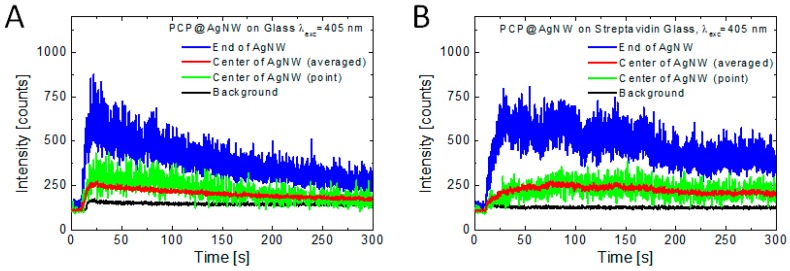
Time-traces of fluorescence intensity measured at the ends (blue lines) and along the AgNWs (red and green lines for averaged and non-averaged data, respectively), as well as background signal level (black lines) measured for the bare glass (**A**) and streptavidin-functionalized glass (**B**) substrates.

The differences in conjugation dynamics and photobleaching behavior taking place for both studied substrates are displayed in Figure 4, where we plot time-traces of fluorescence intensity of selected PCP-AgNWs conjugates. We analyze the behavior of fluorescence intensity along the nanowires as well as at their ends, since as can be seen from the fluorescence maps, the emission at the end is considerably stronger [23]. Furthermore, the time dependence of the fluorescence intensity for spots along the nanowire are analyzed both for a selected spot, as well as for the average intensity along the nanowire, as, similarly to previous experiments, the emission intensity along the nanowire is not very uniform. The intensity variation might be also due to local changes of plasmon enhancement effect or the presence of defects on the nanowires. This might suggest that indeed all the proteins attach to the nanowires and no further PCP complexes are available in the solution. 

At the beginning of the experiment (before dropping the PCP solution), the signal measured for the glass substrate (Figure 4A) is low and constant in time (~130 counts), which is a dark count of the detector. Upon depositing the solution of PCP complexes, at 10 s, rapid increase of the emission is observed at the ends and along the nanowire (blue and green curve, respectively). While the signal in both cases is rather fluctuating, averaging fluorescence intensity over the whole length of the nanowire (excluding the ends), results in much smoother timetrace (red line), although the overall dependence is comparable. After rapid increase of fluorescence intensity, culminated by reaching the maximum value after approximately 20 s, we observe exponential decrease of the emission due to photobleaching. Importantly, the emission signal collected off the nanowire (black line) exhibits qualitatively similar dependence as the emission measured for the PCP complexes conjugated to AgNWs, but its intensity is substantially lower. Based on previous experiments, where no conjugation was applied [23], this difference could be possibly attributed to the plasmon-induced increase of fluorescence intensity of the PCP complexes. Indeed, rough estimation gives a ten-fold enhancement factor of the PCP fluorescence for proteins attached at the ends of AgNWs. 

The situation is qualitatively different, when we consider the results obtained for a glass coverslip functionalized with streptavidin (Figure 4B). While the main effects—increase of fluorescence intensity after dropping PCP solution on a substrate, and the subsequent decrease due to photobleaching—are analogous to the results obtained for a glass substrate. However, the timescale of these processes is visibly different, as both the increase of fluorescence due to conjugation and intensity reduction due to photobleaching are much slower. Also, the correlation between intensity timetraces measured for the ends of AgNWs and along them is less evident than for the nanowires placed on bare glass substrates. Furthermore, there is essentially no signal off the nanowires, as shown in black in Figure 4B. It indicates that due to functionalization of the substrate with streptavidin, and the resulting repulsive interaction with streptavidin-functionalized PCP, no PCP complexes attach to the substrate. In other words, the complexes stay longer time in the solution, making it perhaps more probable for them to find a nanowire and attach to it. Such a process would take longer than in the other studied structure, which might explain longer raise time of PCP emission on AgNWs. The comparison between both substrates, although not all of the details can be fully explained using the collected and analyzed data, implies that the substrate that inhibits conjugation is more suitable for sensing purposes. The increase of fluorescence intensity is in both cases comparable, but the signal to noise ratio is superior for the streptavidin-functionalized substrate. In connection with better photostability, the detection of emission signal is also easier in this case.

**Figure 5:**
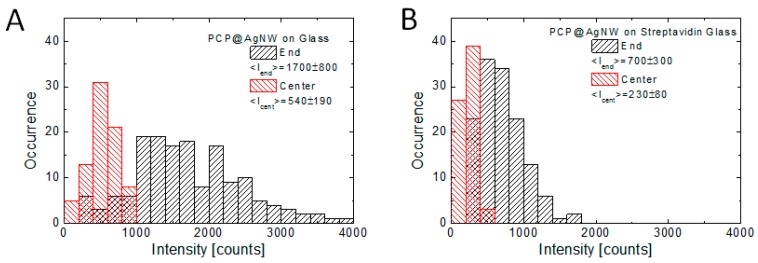
Histogram of fluorescence intensity of PCP complexes conjugated to the ends and centers of AgNWs (black and red bars, respectively) for the bare glass (**A**) and streptavidin-covered glass (**B**) substrates.

The results of fluorescence imaging allow also to compare the effect of plasmon excitations in silver nanowires on the optical properties of the conjugated PCP complexes for both substrates. As can be seen in the fluorescence images, the intensity at the ends of the AgNWs is always higher than along the nanowires. In order to gather sufficient data, we acquired—for both substrates—20 fluorescence images of PCP-AgNWs conjugates, which amounts to approximately 60 single nanowires. The images were taken from the movies and they correspond to the maximum fluorescence intensity, that is to the moment where conjugation was essentially complete and the effect of photobleaching was minimal. All of the maps were measured on areas previously not illuminated. In Figure 5 we show histograms of fluorescence intensities measured for PCP complexes at the ends of AgNWs and along them. The latter values were obtained by calculating the average value for each analyzed nanowire. All values were also normalized to the background level. The results show that on the one hand, the intensities of PCP emission at the ends of AgNWs are substantially higher than those along the AgNWs. The strong plasmonic effect observed at the ends of the nanowires can be due to antennae effect, where higher density of electromagnetic field is expected for structures with high curvature, as well as due to enhanced scattering of plasmons in nanowires at discontinuities [23]. Another possibility would be a higher number of PCP complexes at the curved surface of the nanowire end. On the other hand, the values measured for the substrate functionalized with streptavidin are lower than those obtained for a bare glass substrate. In the case of sample deposited on bare glass the average fluorescence intensities at the ends and along the AgNWs are equal to (1700 ± 600) and (540 ± 190) counts, respectively, while for the sample deposited on streptavidin-functionalized substrate, we obtain (700 ± 300) and (230 ± 60) counts, respectively. The ratio of average fluorescence intensity at the ends and along the AgNWs is equal to 3.15 and 3.04 for the glass and streptavidin-functionalized glass, respectively, which indicates that in spite of differences in actual intensities, the ratio, and thus mechanisms associated with plasmonic interactions in conjugated structures, are similar in these two cases. These values are also similar to previously published results obtained for unspecific mixtures of PCP complexes and silver nanowires [23].

## 4. Conclusions

We describe and apply a method for real-time monitoring of conjugation process between PCP proteins and plasmonically active and appropriately functionalized silver nanowires. It is found-based on wide-field fluorescence imaging—that the conjugation is very efficient as it takes place over a few seconds, as evidenced by emerging bright emission patterns correlated with the positions of the nanowires. Comparative studies of two sample configurations show significant impact of the substrate functionalization on the efficiency of the conjugation process. For the substrate covered with streptavidin the rate of binding the protein to silver nanowires is substantially less than for the bare glass substrate. Importantly, the observed plasmon fluorescence enhancement is independent of the substrate. The described method can be applied for studying protein-nanostructure interactions in similar or analogous structures as they are taking place.

## Supplementary Materials

The following are available online at http://www.mdpi.com/1424-8220/18/1/290/s1, Video S1: Movie of protein attachment to silver nanowires.
